# Implications of healing power and positioning for collaboration between formal mental health services and traditional/alternative medicine: the case of Ghana

**DOI:** 10.1080/16549716.2018.1445333

**Published:** 2018-03-13

**Authors:** Lily Kpobi, Leslie Swartz

**Affiliations:** Department of Psychology, Stellenbosch University, Stellenbosch, South Africa

**Keywords:** Ghana, traditional medicine, collaboration, power, mental health

## Abstract

**Background**: Many current debates about global mental health have increasingly called for collaboration between biomedical and traditional medical health systems. Despite these calls, not much has been written about the variables that would influence such collaboration. To a large extent, collaboration dialogues have considered biomedicine on the one hand, and a wide range of traditional and faith-based treatments on the other hand. However, this dualistic bifurcation does not reflect the plurality of healing systems in operation in many contexts, and the diverse investments that different non-biomedical healing approaches may have in their own power to heal.

**Objective**: We set out to explore the diversity of different healers’ perceptions of power, and the relationship between that power and the perceived power of biomedical approaches.

**Methods**: Through a qualitative design, and using the case of medical pluralism in urban Ghana as an example, we conducted interviews among different categories of traditional and alternative medicine (TAM) practitioners living and/or working in the Greater Accra Region of Ghana.

**Results**: Through thematic analyses, differences in the notions about collaboration between the different categories of healers were identified. Their perceptions of whether collaboration would be beneficial seemed, from this study, to co-occur with their perceptions of their own power.

**Conclusions**: We suggest that an important way to move debates forward about collaboration amongst different sectors is to examine the notions of power and positioning of different categories of TAM healers in relation to biomedicine, and the attendant implications of those notions for integrative mental healthcare.

## Background

In many low- and middle-income countries (LMICs), access to and use of formal mental health services is limited for various reasons, including shortage of trained professionals, limited resources and perceived high cost of care [1–3]. As a result of these and other factors, alternative and complementary healthcare methods such as traditional and faith healing are popular avenues for receiving care in many LMICs, including many African countries [4–7].

Some previous studies have explored the use of traditional and alternative medicine (TAM) by patients and caregivers of people living with mental illness in different African contexts [6–11]. These small-scale studies have argued that generally, patients and caregivers seek the services of TAM practitioners because they are more easily accessible and often more flexible in terms of payment structures, but also because their values, concepts and beliefs are similar to those of the patients. Therefore, there was the inclination for service users to seek their services first. Even for those who did not seek traditional remedies as a first point of call, the strong side effects of psychotropic medications often made them undesirable for continued use [12].

Other lines of research have examined the beliefs that are held by TAM practitioners about mental disorders [13–18]. In these studies, the prevailing notion about causation was supernatural in nature. That is, traditional/faith healers generally believed that evil spirits, demonic possession, curses and spiritual punishment manifested as mental disorders. Even though their views were dominated by supernatural factors, many of the healers did acknowledge that other factors such as drug misuse and traumatic brain injury were possible causes of mental disorders.

In addition to their causal beliefs, there have also been studies on how TAM practitioners treat mental illness [19–24]. The treatments varied based on the orientation of the healer. The common treatments reported included herbal remedies (such as infusions, decoctions, inhalants and ointments), dietary restrictions, psychosocial counselling, prayers and incantations, among others.

In current debates about global mental health, it has become commonplace to call for a closer collaboration between biomedical, or psychiatric, approaches to treatment, on the one hand, and a wide range of faith-based practices on the other, given the popularity of alternative treatments in many LMICs. There have been some examples in the literature of (generally small-scale) attempts at collaboration between mental health professionals and traditional healers [25–31], but, though the call for collaboration continues as it has done for many years, it is somewhat surprising that there have not been more studies on how collaboration may or may not work.

The World Health Organization (WHO) strategy document on traditional medicine [32], as well as its mental health action plan [33], acknowledges the need to recognise the diversity that exists in traditional and alternative treatment options, and advocate for country-specific strategies to be developed based on contextual needs. However, there is a strong emphasis on co-operation and regulation of TAM practitioners along the medical model. The recommendations are fundamentally for biomedicine to provide pharmaceutical care while TAM provides complementary care along psychosocial and spiritual lines. They also advocate for more research to be done on the quality, effectiveness and forms of TAM, taking into account the environmental and social as well as spiritual factors which make up TAM approaches to healing [34].

Despite these acknowledgements, the dualistic bifurcation between western medicine on the one hand and traditional/faith healing on the other does not reflect the plurality of healing systems in operation in many contexts – there are many different kinds of healers, using differing systems of justification for their work, and engaging in complex and at times unpredictable ways [35]. Consequently, part of what has not been fully explored in the study of the potential for collaboration between western medicine and other approaches to healing is the question of the diverse investments that different non-biomedical healing approaches may have in their own power to heal, and the relationship between that power and the perceived power of biomedical approaches. Using the case of medical pluralism in urban Ghana as an example, in this paper we argue that an important way to move debates forward about collaboration amongst different sectors is to examine the notions of power and positioning of TAM healers in relation to biomedicine.

## The case of Ghana

As part of a larger study, we conducted interviews among different categories of TAM practitioners living and/or working in the Greater Accra Region of Ghana. For ease of presentation, we have used four categories of practitioners; we do, however, acknowledge that the Christian, Muslim and indigenous African religious healers may be classified collectively as faith healers. Thirty-six practitioners were interviewed, made up of 8 herbalists, 10 Islamic healers, 10 Pentecostal/charismatic Christian faith healers and 8 traditional shrine priests/medicine-men (see Table 1 below for a summary of the demographic characteristics of the participants). In other papers, we have discussed in more detail the work of each of these groups of healers (for herbalists’ methods, see [20]; separate manuscripts for Christian faith healers, Muslim faith healers, and shrine priests are currently under consideration elsewhere).

**Table 1.** Summary of demographic characteristics of participants.

Characteristic	Number (%)
Gender	
*Female*	5 (13.9%)
*Male*	31 (86.1%)
Type of healer	
*Herbalist*	8 (22.2%)
*Shrine priest*	8 (22.2%)
*Mallam*	10 (27.8%)
*Pastor*	10 (27.8%)
Mean age	54.6 years
Mean years of practice	28.1 years

### Pentecostal/charismatic Christian healers

The Christian faith healers all subscribed to the Pentecostal/charismatic doctrine of Christianity which places much emphasis on prophecies, miracles and the gifts of the Holy Spirit [36]. These pastors claimed that they had received special gifts of healing from God, through which they performed miracles of healing for people with various ailments. They set up healing centres (called prayer camps), which were often filled with patients and their caregivers seeking divine intervention for their illness. Some of the camps offered housing for patients and their caregivers, while they sought healing from God. Their healing methods included prayer, fasting and exorcism. Some pastors used these methods alone while others combined them with prayer aids such as holy water and anointing oil. Some pastors advertised their services through radio and television programmes, billboards and posters. In addition to these, witness testimony was an important means of creating awareness of the camps.

From our interviews, the pastors considered themselves to be operating at a higher level of efficacy than biomedical professionals. They considered their methods to produce more enduring results given their use of the gifts of the Holy Spirit, whom they considered all-powerful. They demanded respect and reverence, and expected their instructions to be followed closely in order for the patient to receive complete healing. Despite the self-perception of power that this expectation of obedience may suggest, there was a strong desire among the pastors to be formally recognised for their work and abilities. Many of them envisioned a system in which they worked alongside doctors to provide services to patients in hospitals. As one pastor put it, ‘they have their area – which is the physical side – and we … handle the spiritual side’. This was said to emphasise the need for recognition and collaboration with the formal health system. Thus, despite their assertion that their methods worked better than biomedical methods, they acknowledged the place of biomedicine. They also perceived biomedicine to have greater recognition and respect in the national health discourse, and, by extension, greater power and legitimacy in the eyes of the government.

### Muslim healers (mallams)

The Muslim healers were learned Islamic clerics who had been trained in how to apply the words of the Qur’an and other Islamic texts like the Hadith in treating various illnesses. Some had received further training to incorporate plants and animal parts in the healing process. Those who incorporated herbs in their healing work held informal clinics on specific days where patients requiring the combined therapy could be brought for care. These were also the healers who used posters, billboards and radio to advertise their services. However, those who relied solely on the Qur’an were typically leaders of local mosques and did not advertise their services. The Muslim healers, called mallams in local parlance, were all male.

Similar to the pastors, the mallams viewed their healing as being more efficacious than biomedicine, in their case, due to their use of the words of Allah and his prophet. Unlike the pastors, however, they did not ascribe any power to themselves and constantly emphasised their position as servants of God in the work of healing. According to them, to take credit for the outcomes of their work would be inappropriate given that their role in the healing process was to recite the words that they had been given – words which contained the power to restore health to the patients. They did not desire association with biomedical professionals because they believed the two systems functioned on different planes. This is not to suggest that they were opposed to biomedicine completely, but rather their belief was that disorders which they could treat were not physiological in nature and hence did not require the intervention of doctors. Yet they also believed that doctors had been given wisdom by God to treat physiological problems. To them, each system of care had its place, and both were necessary for the complete well-being of the patient.

### Shrine priests

The shrine priests, or medicine-men, were devotees of indigenous African deities. These traditional religious healers represented and carried out the wishes of various deities or gods. Their shrines were typically located in remote, isolated areas such as groves. Through their association with the gods, they divined the nature and causes of whatever disorder the patient presented with. Their healing methods depended on the directions received from the gods and could involve actions to be undertaken by both the patient and their family. In some cases, the healers used herbal remedies to supplement the spiritual intervention.

The shrine priests expressed similar sentiments as the mallams regarding their own power. These healers also viewed themselves as conduits for the gods that they represented. They did not ascribe any supremacy to themselves; however, they considered themselves powerful as agents of the gods whom they served. Given that power, they demanded fear and reverence be shown to them as befits the gods’ status. On the other hand, this also suggests that they believed they bore no responsibility for the consequences of their actions, given that they were relaying the wishes of a higher power. Despite this perceived ambiguity, they always emphasised their obligation to not harm the patients, an action which they believed would result in dire consequences for them. The shrine priests sought no recognition from biomedicine, and showed no drive for legitimacy because, as one priest indicated, ‘Whether they work with us or they don’t work with us, [the god] will still be powerful.’ Thus, they were powerful only by virtue of their association with the powerful deity. As such, they did not require recognition from formal bodies to know their worth and abilities.

### Herbalists

The last group of healers, the herbalists, considered themselves scientists who harnessed the properties of herbs and plants to heal patients. Some of them had established herbal clinics where patients came for consultation and where they produced various tonics and ointments. Others sold their herbal remedies on buses and in marketplaces. Many of them advertised their goods through media such as posters and billboards, as well as radio and television advertisements. Although most of them treated a wide variety of sicknesses, some of them indicated that they had specialised in treating mental disorders.

The herbalists viewed themselves as ‘work[ing] the same way the doctors do’. By this they meant having an affinity with the systematic methods of diagnosis and treatment used by biomedicine. However, many of them repeatedly emphasised the fact that they were using time-tested methods that had been handed down from their ancestors, unlike the ‘white man’s system’ which was used by conventional doctors. According to them, these herbal methods were developed within the indigenous cultural context of the targeted people, and as such served a greater purpose than simply ridding the patient of symptoms. Ironically, this notion was held even by herbalists who self-identified as Christian or Muslim. As a result of this view, many of them included an aspect of spirituality in their treatment regimens. It was therefore common to have treatment programmes which included prayer and fasting, or the recitation of incantations.

By asserting that their methods were culturally sensitive, yet systematic as in biomedicine, the herbalists appeared to occupy a position of liminality [37] within the field of global pharmaceutics. That is, they situated themselves between biomedicine and indigenous knowledge, providing a more holistic, more affordable and easily accessible service which was built on an understanding of cultural values and ideas [38–41], added to an appreciation for the methodical nature of modern medicine. Consequently, they believed this afforded them greater power for healing. However, the herbalists sought collaboration with biomedical professionals, perhaps as a way of proving their legitimacy and asserting their influence in healthcare.

## Discussion

For any healthcare system, the extent to which the methods are considered powerful for treating specific conditions is influenced by perceptions of efficacy and effectiveness of the beliefs and practices employed by that system [42]. This notion of power is not limited to the ability to prescribe/produce appropriate medication (whether biomedical or herbal), but also suggests an ability to recognise and identify the causal elements of a sickness [12,43]. Thus, a biomedical practitioner who prescribes psychotropic medications which ‘cure’ a patient’s physical disorder may be considered just as powerful as the pastor or shrine priest who is able to discern witchcraft as the cause of a spiritual disorder and perform an effective exorcism. The two may be considered equally powerful, yet operating in parallel dimensions.

When illness is conceived as a punishment or the consequence of some moral failing, the search for healing may be directed towards aligning with a source of moral power [44]. This source of power is typically reflected in the work of religious healers. However, the assertion that certain physiological processes can be present in the body of one who is mentally ill would result in an alignment with so-called physical remedies in the form of psychotropic medication or herbal remedies. At the end of the day, patients search for the treatment option which will bring relief from their ailment.

Thus, the perceived efficacy of the treatment is echoed in the perceived power of the healer to cure the ailment. At the core of these notions of power lies an expectation that the outcome of treatment will be a complete cure of the disorder [12,35]. This cure is manifested when the treatment restores patients to their previous state of productivity, and they are able to reintegrate into the social strata of the community. Therefore, patients’ search for healing would not rest solely on identification with a particular healing system. Instead, they would utilise the system which in their view yields the desired cure. Similarly, healers measure their power and authority over illness in relation to the capacity of their methods to cure illness.

Given this premise, for any efforts to collaborate and scale up mental healthcare in African countries to succeed, the strategies for TAM must not ignore the illness beliefs of the populace. They must also appreciate the real challenges (such as the strong side effects of psychotropic medication) that exist for patients and their families in the use of biomedicine [12].

But a further consideration would be an appreciation of the diversity that exists in the relationship between the various healers’ claims to power and the power they see afforded by biomedical approaches, and, by so doing, revising the dualistic view of health-seeking. The recent movement for global mental health advocates the development of standard packages of care as a way of affording universal western psychiatric care, particularly in LMICs [45]. Again, such goals need to be situated within the pluralistic framework of healthcare in these countries, and cannot overlook the scientific uncertainties around aetiology and course of mental disorders [12], as well as the competing notions of power that exist among the various categories of healers.

In this article, we have made a small first step in exploring the diversity of power claims made by different sorts of healers. To suggest to biomedical practitioners that they should collaborate with TAM systems is important given resource constraints, but it is clear that the bases for collaboration with different kinds of healers may be different. In our study, the Pentecostal Christian healers and herbalists were more desirous of working with biomedicine, whereas Muslim healers and shrine priests were less interested in collaborating. Interestingly enough, it is also the Pentecostal/charismatic pastors and the herbalists who are more closely positioned in relation to the formal economy. Pentecostal and charismatic churches are hugely popular and financially profitable in Ghana and other African countries [46,47], and it is possible that collaboration with biomedicine could extend the power of the already powerful and lucrative church practices.

Similarly, herbalists operate in a lucrative global pharmaceutics market [38,39] and could also gain by being part of a referral network with biomedicine. By contrast, both Muslim healers and shrine priests operate on smaller and more local scales and appear to have less to gain from collaborating with biomedicine. They do not see themselves as powerful but as instruments of spiritual power. Alternatively, their reluctance to integrate into the mainstream health system may be as a result of their reluctance to lose their position of prominence in contexts where respect for biomedicine dominates perceptions.

Two points of caution are necessary here. First, our data come from a relatively small sample, and it is clear that much more work needs to be done to tease out the potential complexities of collaboration by biomedicine with healers of different kinds. Second, we do not wish to suggest that the reasons Christian healers and herbalists in our sample were interested in collaborating with biomedicine were mercenary and purely self-serving. We are suggesting simply that questions of the benefits of collaboration amongst health systems must be considered not only in terms of potential patient welfare, but also in terms of whether there are perceived advantages to different healers to collaborate. In our study, different types of healers were positioned differently in terms of this question, and the gradient of perceived benefit to the healers seemed, from this small study, to co-occur with the perceptions by healers themselves of their own power. Clearly, more work on this question needs to be undertaken.

## Conclusions

The WHO and other bodies have called for collaboration in mental health between biomedical practitioners and TAM, for a range of good reasons. Given its widespread use in LMICs, as well as the popularity and cultural relevance of TAM among minorities in high-income countries, it is important that an in-depth understanding of all facets of these systems of healthcare be understood in order to achieve the desired integration [48,49]. From the discussions with our participants, it is clear that TAM is not an undifferentiated field. There are some similarities across different healing sectors regarding illness beliefs; however, the perceptions of practitioners’ understandings of their own role and power show some variation.

These differences may well be important for collaborative efforts. Specifically, in our study, it appears that the healers who considered themselves to be most powerful were most willing to work with other health systems. On the other hand, the Islamic and shrine healers, who insisted on not taking credit for the health outcomes of their patients, were less desirous of working with biomedical healers to treat mental disorders. This suggests, perhaps, that the eagerness to collaborate may in part be a move towards achieving legitimacy and recognition, as perceived to be held by the biomedical field.

These different notions of place also reflect different collaborative models held by the healers [50]. The pastors’ eagerness to work alongside biomedical practitioners may be a reflection of their endorsing an incorporation of TAM with biomedicine, where aspects of each paradigm are selectively utilised for patient care. However, the disinterest of the mallams and shrine priests, as well as the ambivalence of the herbalists, is reflective of the pluralisation model, where each remains largely independent of each other while acknowledging the service users’ right to choose treatment options.

The important question for integrative healthcare systems must therefore be more nuanced than simply a call for collaboration. In Ghana, and likely in other countries, we need to know more about who, and from which groups, would wish to work together for mental health, and for which reasons. Questions of place, power and claims to legitimacy may form an important component of the collaboration dialogue. Collaborative efforts, we suggest, may be less likely to succeed if these contextual factors regarding different types of healers are not considered. There is clearly still a great deal of work to be done in this area.

## Paper context

Recently, there have been calls for integration of different mental healthcare systems in LMICs. These calls do not appear to consider the different views on collaboration which may exist among different categories of traditional/faith healers, based on their perceptions of their power to heal. We examine this diversity by analysing medical pluralism in Ghana, and suggest that questions of place, power and claims to legitimacy should form an important component of the collaboration dialogue.
